# Mechanics of Constriction during Cell Division: A Variational Approach

**DOI:** 10.1371/journal.pone.0069750

**Published:** 2013-08-21

**Authors:** Victor G. Almendro-Vedia, Francisco Monroy, Francisco J. Cao

**Affiliations:** 1 Departamento de Física Atómica, Molecular y Nuclear and Departamento de Química Física I, Universidad Complutense, Avenida Complutense s/n, Madrid, Spain; 2 Departamento de Química Física I, Universidad Complutense, Avenida Complutense s/n, Madrid, Spain; 3 Departamento de Física Atómica, Molecular y Nuclear, Universidad Complutense, Avenida Complutense s/n, Madrid, Spain; Université de Genève, Switzerland

## Abstract

During symmetric division cells undergo large constriction deformations at a stable midcell site. Using a variational approach, we investigate the mechanical route for symmetric constriction by computing the bending energy of deformed vesicles with rotational symmetry. Forces required for constriction are explicitly computed at constant area and constant volume, and their values are found to be determined by cell size and bending modulus. For cell-sized vesicles, considering typical bending modulus of 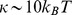, we calculate constriction forces in the range 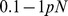. The instability of symmetrical constriction is shown and quantified with a characteristic coefficient of the order of 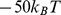, thus evidencing that cells need a robust mechanism to stabilize constriction at midcell.

## Introduction

Cell constriction is an important cytokinetic phase preceding division. Before splitting in two daughters, symmetrically dividing cells accommodate theirs duplicated contents into spatially separated compartments defined by a stable fission site located at midcell [1]. Constriction is a non-spontaneous process which involves large membrane deformations at the site of fission, a division route entailing a strong breakage of symmetry in the mother cell. In many organisms, membrane constriction is mediated by a ring-shaped centripetal apparatus able to stress a radial force in the constriction site [1], [2]. Quite a lot of energy is required to distort the equilibrium shape into a transitory constricted configuration potentially able to finally lead to binary fission [3]. This is a crucial problem of bioenergetics, whose solution should inform us about the amount of mechanical energy necessary to duplicate cell contents in a stable way [4], [5], [6]. Computing the constriction forces requires a knowledge about the minimal energy configuration at each constriction stage. Different methods have been proposed to obtain the minimum energy shape of a membrane under given constraints [7], [8], [9]. They consider numerical procedures [7] and perturbative approaches [9], including explicit structural characteristics (vesicle topology, spontaneous curvature, membrane tension and excess area) within a mechanical kernel defined by the Canham-Helfrich Hamiltonian of the membrane bending elasticity [3], [10], [11], [12]. However, despite these detailed descriptions, a simplified approach to the problem of the spherical binary fission is still lacking. Here, the question is addressed by considering a spherical vesicle under symmetric constriction up to final fission into two daughter cells. In order to compute the minimum energy shapes we propose a variational approach to the vesicle shapes along the constriction pathway. The variational problem is resolved using a minimal mechanical kernel based on the bending energies considered under the simplest conditions. First, we will assume a homogeneous membrane with an average zero spontaneous curvature, a condition globally fulfilled by the planar lipid bilayer assembly which reasonably represents the simplest model of a biological membrane [3], [13]. Then, the membrane is considered in an initial spherical configuration which is the most stable tensionless shape compatible with a zero excess area. No tension effects are considered so far, a condition accounting for the different mechanisms of membrane biogenesis existing in cells. In order to account for constriction, a blunt profile is proposed as a variational ansatz describing membrane shapes at the constriction region. The results are discussed for different ideal cases accounting for given constraints. Namely, constant area and constant volume will be considered. The paper is organized as follows: In Sec. 1 we present the model used to compute the bending energy and its simplification to the case of surfaces of revolution. At the end of that section, we introduce the variational approach used to compute the minimum bending energy shape. In Sec. 2.1 we compute, for symmetric constriction, the minimal bending energy and its corresponding shape for different constriction stages. Section 2.2 focuses on the constriction force needed along the constriction pathway. Next, in Sec. 2.3, we study and quantify the stability conditions for symmetrical constriction. This quantification allows to establish the minimum effective potential required to stabilize symmetrical fission under axysymmetric constriction. Finally, in Sec. 3, we conclude by summarizing the conclusions.

## 1 Methods

### 1.1 Elastic Hamiltonian: Bending energy for surfaces of revolution

Changing the shape of a spherical vesicle from its equilibrium configuration is a non-spontaneous process that requires an input of energy. In the minimal description, the energy of the vesicle shape changes is assumed to exclusively involve bending elasticity, particularly, contributions from mean and Gaussian curvatures [3]

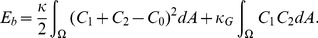
(1)In this equation, 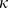 is the bending modulus, 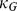 the Gaussian bending rigidity, 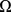 the surface, 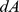 its element of area, 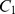 and 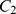 its principal curvatures, and the parameter 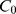 is the spontaneous curvature that effectively accounts for possible asymmetries in the surface structure between the inner and the outer sides. Here, we restrict ourselves to the simplest case 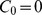, corresponding to a lipid membrane without the elements of structural complexity necessary to support spontaneous curvature (the membrane is flat in its absolute minimum energy configuration). The integrated Gaussian curvature, the second term in Eq. (1), is invariant under shape changes that do not change topology [3], [14]. The constriction process does not change the topology, and only involves shapes that are topologically equivalent to a sphere. For the sphere, the bending energies are 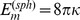 and 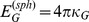, respectively for the mean and Gaussian contributions. Therefore, although we describe the Gaussian curvature, we will only deal with the variations of energy due to the mean curvature
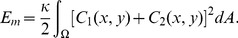
(2)When the surface can be represented in Cartesian coordinates as 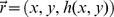.

**Figure 1:**
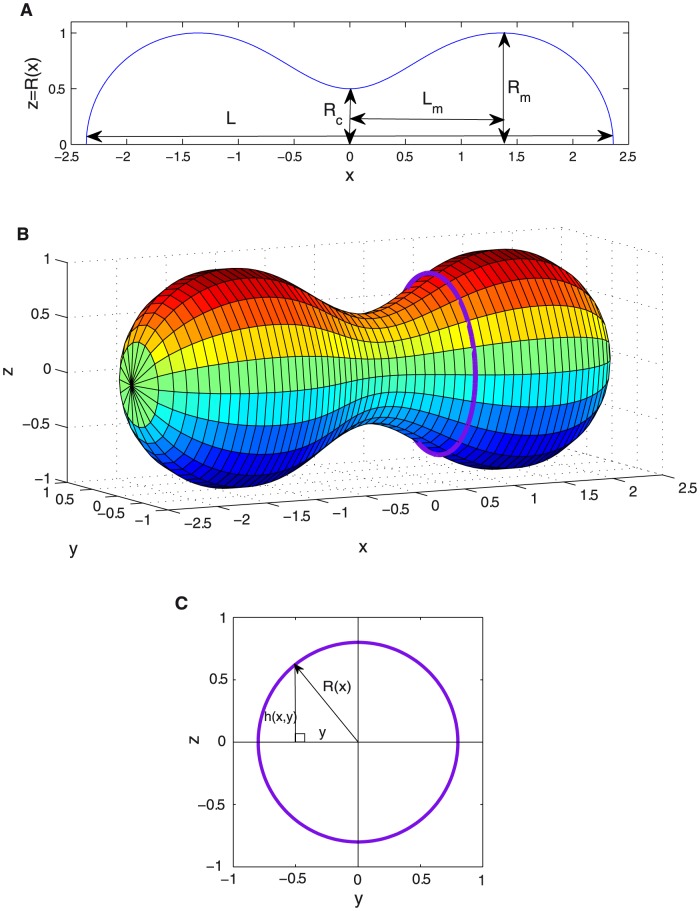
Symmetrically constricted vesicle. A. Longitudinal section at 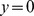 and characteristic parameters of a deformed vesicle under symmetrical constriction represented on the optimal shape obtained for 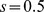 using first order approach. B. Surface resulting from the revolution of the optimal shape represented in Fig. 1A. C. Transversal section at a given 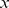. The height at a given point 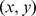 is given by 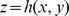. Due to rotational symmetry around 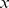 axis, all transversal sections are circumferences. We denote its radius by 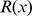. The height 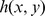 and the radius 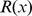 are related by the Pythagoras' Theorem 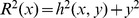 which leads to Eq. (3).

For a surface of revolution with rotation symmetry axis along 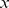,
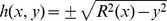
(3)parameterizes the surface, with 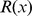 the radius of the section of the surface at 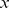 (see Fig. 1). If the surface is between 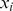 and 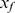, its total area would be
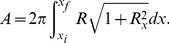
(4)For the total volume enclosed by the surface we have
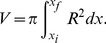
(5)Analogously, once the membrane profile is known, the bending energy 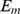 [Eq. (2)] for a surface of revolution is given by
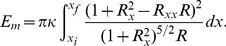
(6)(See Supporting Information for details on the derivation of the previous equations.) The scale invariance of the bending Hamiltonian in Eq. (6) for surfaces of revolution implies no dependence of the bending energy on the system size. Thus, in Eq. (6), for any shape the transformed under the overall dilatation 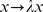 and 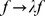 has the same bending energy (see Supporting Information for details). This implies that once we have determined the shape that minimizes the energy, its transformed under an overall dilatation has the same energy and also minimizes the energy. This property will be very useful for us in this paper. It will be also useful to recall that under an overall dilatation the area is transformed as 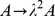 and the volume as 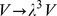. The vesicle takes the shape that minimizes this bending energy 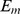 (up to thermal effects).

### 1.2 Variational approach to minimization

The procedure considered in this paper consists to find the shape that minimizes the bending energy using the variational method [15]. This method is based on the fact that all possible shapes give energies greater or equal than the global minimum, and the shapes that gives energies equal to the minimum are the optimal shapes [16]. In order to apply the procedure, we will consider a family of shapes of the form
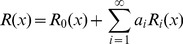
(7)where 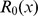 and 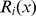 constitute a complete basis of functions which will cover all possible shapes under this description. It is important to recall that any truncation of the series is a variational ansatz, which is expected to satisfy four boundary conditions at two extremal points (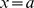 and 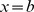), namely, 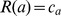, 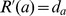, 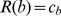, and 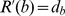. We will choose a zero-th order solution 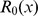 that verifies the same boundary conditions as 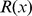 [i.e., 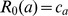, 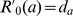, 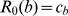, and 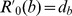]; while the family of functions 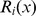 are chosen to fulfil zero boundary conditions, i.e., 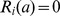, 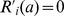, 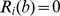, and 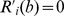. In this way, boundary conditions are guaranteed for all members of the family of functions (all values of 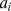). In addition, we would try to choose in practice the functions 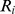 in such a way that, for the optimal shape, the coefficients 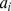 rapidly decrease with increasing 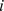. In that case, keeping the first few terms of the series we can arrive at a good approximation both for optimal shape and for minimal bending energy. In summary, this leads to the following practical procedure: choose a softly varying 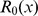 that satisfies the boundary conditions for 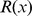, choose a complete set of functions 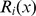 (independent of 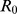) that satisfies the analogous zero boundary conditions, order the set from softly varying (lower energy for the same amplitude, thus higher amplitude expected) to more abruptly varying, keep the first terms of the set of functions and minimize bending energy with them, iteratively include more functions 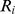 in the minimization to improve the approximation and estimate the error. Although the method is restricted here to the case of surfaces of revolution, it can be easily extended to general surfaces.

## 2 Results and Discussion

### 2.1 Minimal bending energy and optimal shape at a given constriction stage

Scale invariance of the bending energy tell us that bending energy of a vesicle is independent of the total area of its surface (or of the total volume enclosed) and only depends on the shape of the vesicle. Thus, we will start by finding the vesicle shapes that minimize the energy for a given constriction stage. The constriction stage will be characterized by the constriction parameter
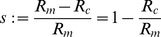
(8)which is defined in terms of the ratio of the constriction radius 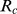 to the maximum radius 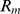 (see Fig. 1a). During the constriction process this dimensionless parameter goes from 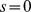 (no constriction) up to 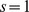 (maximal constriction).

First, we will consider solutions at constant 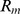, which will be discussed as a reference state for other conditions as fixed area or volume, computed using a rescaling procedure described in the next subsections.

#### 2.1.1 Constant maximum radius 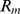


We consider vesicles shapes with a constricted central region analogous to the one depicted in Fig. 1b, i.e., with revolution symmetry along a longitudinal axis and with central symmetry. This shapes are generated rotating, along the 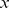 axis, profiles 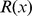 as the one depicted in Fig. 1a, central symmetry implies 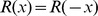. Then, we divide the profile 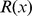 in four regions separated by the three zeros of its derivative 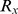, located at 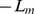, 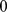, and 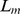, and search which is the profile that minimizes the bending energy at a given constriction stage 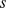. Now, in terms of functions, its components are 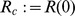 and 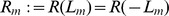.

**Figure 2:**
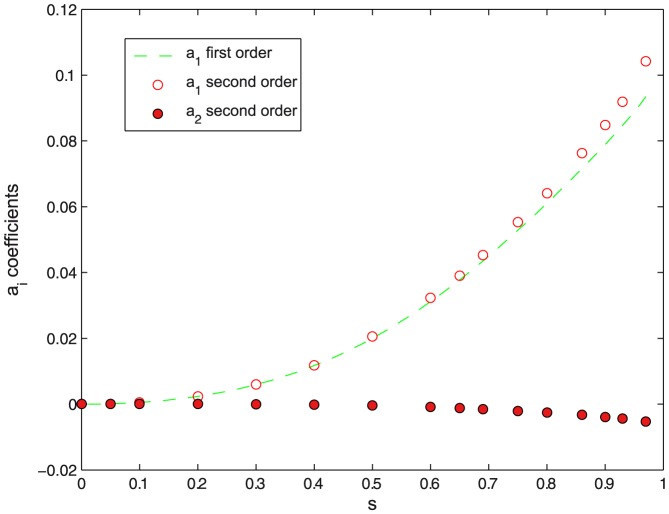
Values of the variational parameters 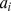. Values of the parameter 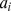 as a function of constriction parameter 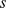 for different orders of approximation in the variational approach.

**Figure 3:**
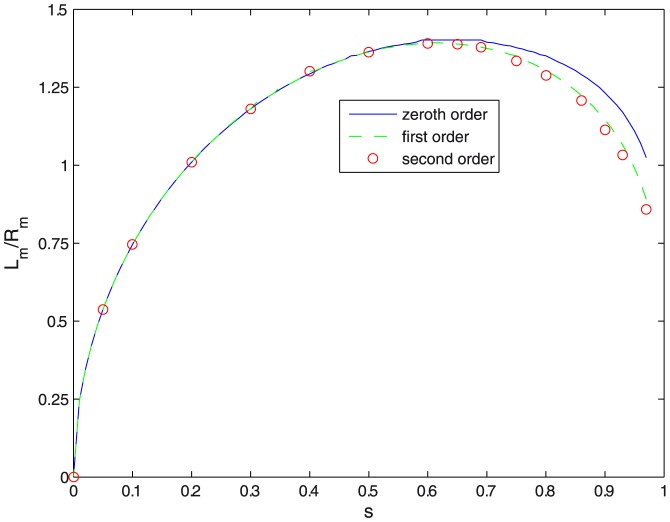
Aspect ratio of the constriction region. Aspect ratio of the constriction region 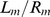 as a function of constriction parameter 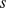 for different orders of approximation in the variational approach.

**Figure 4:**
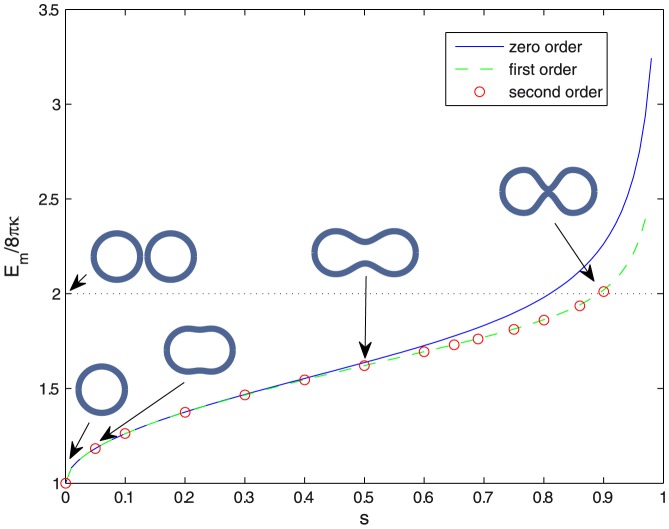
Bending energy. Bending energy 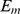 (in units of 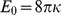) as a function of constriction parameter 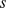 for different orders of approximation in the variational approach. Profiles maintaining constant 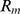 at different stages of constriction are also shown.

**Figure 5:**
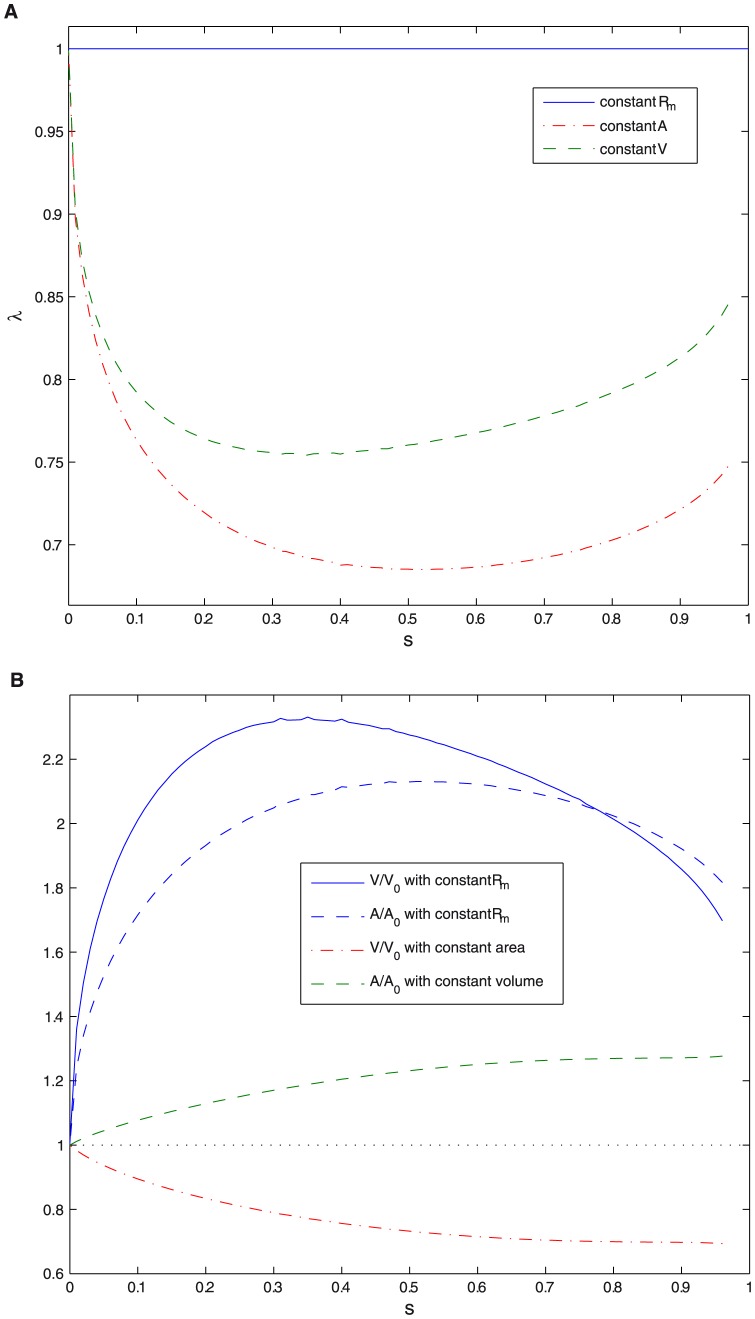
Rescaling parameter 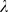 , area and volume. A. Rescaling parameter 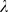 as a function of constriction parameter 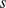, for different cases: constant maximum radius, constant area, and constant volume. B. Area 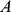 in units of 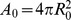 and volume 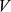 in units of 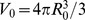 as a function of constriction parameter 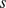 for different constraints.

The rightmost polar region has boundary conditions 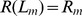, 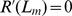, 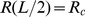, and 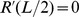, with 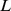 the total longitudinal length of the vesicle (see Fig. 1). For the polar regions, the shape during constriction continues to be close to a hemisphere, i.e. a half-dome, of radius 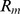. This correspond to 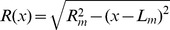 for the right pole, and to 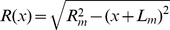 for the left pole. Therefore, the total longitudinal length of the vesicle is 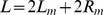. These considerations leads to
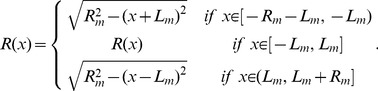
(9)where only the shape in the central constriction region remains to be determined. The shape 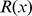 with symmetry 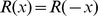 that minimizes the energy in this central region has to verify the boundary conditions 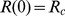, 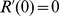, 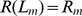, and 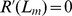. We determine the optimal shape 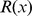 in the constriction region applying the variational approach that we have previously presented. Therefore, we will consider families of solutions of the form
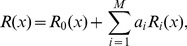
(10)with 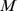 the order of the approximation. We take
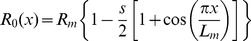
(11)

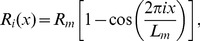
(12)where 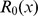 verifies the boundary conditions 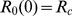, 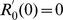, 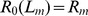 and 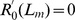; and all 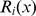 the analogous zero boundary conditions: 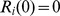, 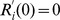, 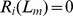, and 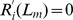. Bending energy increases with increasing curvature, which correspond to larger values of the derivatives of 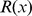. The family of functions considered constitutes a complete basis and it is ordered form slower varying function (less energetic shapes) to faster varying functions (more energetic shapes). Therefore, we expect that the coefficients 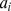 decrease fast as 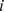 increases for the optimal shape. Applying the variational approach we determine for each constriction stage the values of the shape parameters 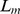 and 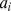 that minimize the bending energy along the constriction pathway where 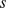 is continuously increased (with 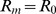, being the radius of the initial sphere). These results allow us to compute relevant magnitudes as a function of the length 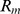 and the bending modulus 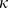 at different constriction stages. Figures 2–4 show that the convergence is fast for the families of functions chosen. The amplitudes of the variational parameters 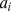 and 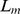 are plotted in Figs. 2 and 3 respectively, up to second variational order, which provides an accurate approximation in the constriction region. Third- or higher order represents very small contributions characterised by negligible values of the corresponding variational parameters. Indeed, at small constriction, the minimal energy profile is reasonably described by the zero-th order function in Eq. (10). Only at large constriction (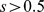), small contributions from the first order variational terms are required to describe the profile of minimal energy (see Fig. 2). The relative contribution of the second order variational term is comparatively negligible (see Fig. 2), making the first order approach sufficient to make converge the bending energies down to their lowest variational minima. Figure 3 shows the 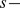
dependence of the optimal aspect ratio of the constriction region 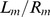. At zero constriction (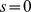) the aspect ratio takes a null value, corresponding to the initial spherical case (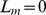). Increasing constriction causes the vesicle to axially elongate the constriction region, with 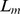 increasing until it reaches a maximal value 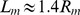 at a constriction 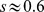. Further constriction beyond maximal elongation (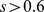) causes a little contraction down to a value compatible with unity (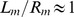), which resemble two identical spheres joined by a narrow neck. Figure 4 shows constriction is a non-spontaneous process requiring an input of energy to occur. The reduced value of the bending energy 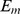 increases monotonically with increasing 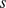, starting at 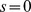 from the value corresponding to the initial sphere, 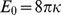. At high constriction (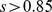), the bending energy exceeds 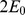, the final bending energy corresponding to the two spheres representative of the fissioned state (see Fig. 4). Such an excess energy 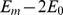 represents one of the contributions to the energy barrier 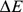 between the pre-fissioned configuration with two spherical lobes connected by a highly curved neck and the final state represented by two separated daughter vesicles. The other contribution is due to the increase in the Gaussian curvature of the two spheres, whose value is 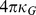. This gives an energy barrier for fission of
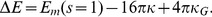
(13)Let's first consider constriction maintaining a constant maximum radius 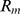. This corresponds to a case where the constriction region is a neck tube between two spherical poles with fixed radius, and the vesicle can change both area and volume unexpensively. Here, Fig. 4 shows the evolution of the vesicle profiles from the initial spherical state at 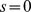 up to the final highly constricted state along the minimal energy constriction pathway defined by the variational amplitudes in Fig. 2 and the aspect ratio of Fig. 3. Forcing constant 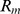 makes both area and volume more than twice their initial values (see Fig. 5b).

A more realistic calculation should consider a variable maximum radius 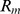 under a given geometrical constraint, e.g. constant area or volume. However, scale invariance makes that the results for 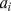, 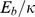 and 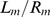 are valid independently on whether we are considering fixed maximum radius 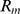 or fixed area 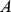, or volume 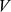, provided we consider the same constriction stage (given by the constriction parameter 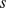).

#### 2.1.2 Constant area vs. constant volume

In the previous section, we have computed the optimal shape that minimizes the bending energy for different constriction stages considering constant maximal radius, i.e., 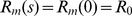. Here, we will address constriction with other relevant fixed parameters, as fixed area or fixed volume. Thanks to the scale invariance of the bending energy solving these problems using the previous results is a simple task. Scale invariance allows us to rescale solutions through appropriate overall dilatations to fit other constraints. As in all cases we start (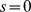) with a sphere of radius 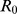, we have that constriction at constant area or constant volume is obtained rescaling the results for fixed 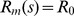 by an overall dilation factor that depends on the constriction parameter
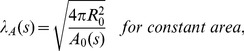
(14)or
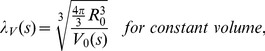
(15)where 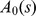 and 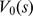 are the area and volume found in the previous subsection for the case of fixed maximum radius 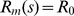. Thus, for constant area 
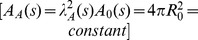
, the maximum radius is 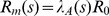, and the volume is 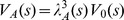. Analogously for constant volume 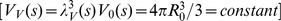, 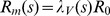 and the area is 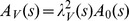. The dilation factors are plotted in Fig. 5a as a function of constriction parameter 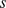. This procedure allows to obtain the optimal dimensions at various constriction stages of a vesicle that undergoes constriction while keeping constant either its membrane area or its internal volume. Figure 5b shows constant volume constriction involves an increase in membrane area. Conversely, constriction at constant membrane area requires a decrease in volume.

**Figure 6:**
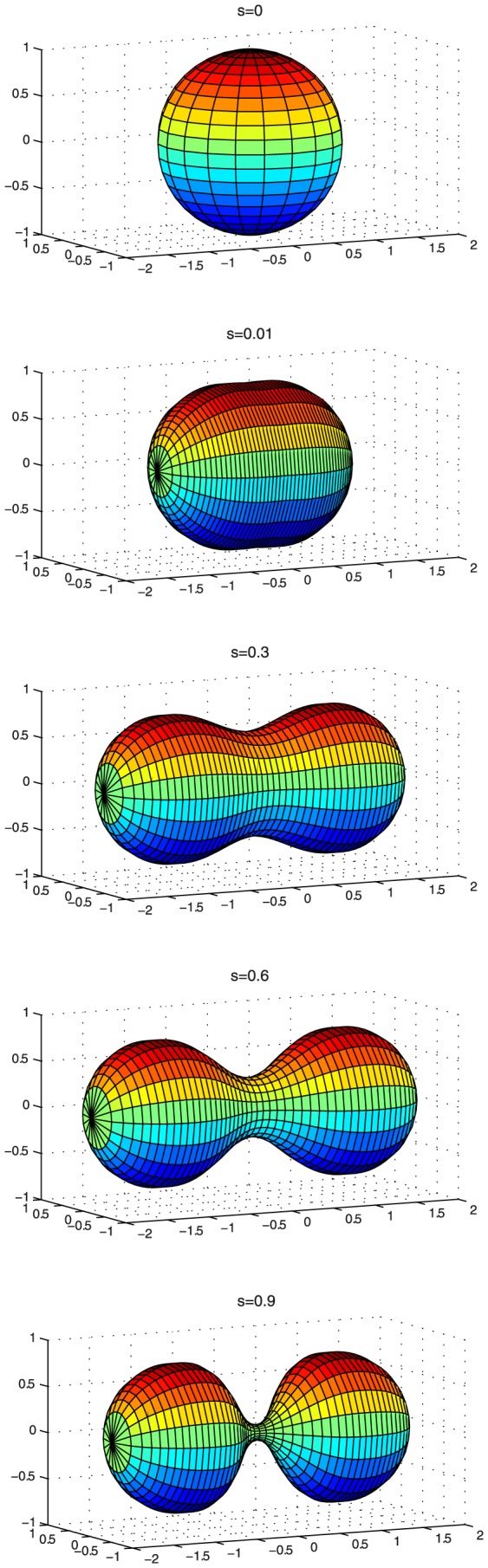
Shapes during constriction process. Shapes at various constriction stages (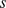 = 0, 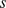 = 0.01, 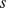 = 0.3, 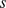 = 0.6 and 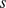 = 0.9) with the condition of constant volume.

Constant volume constriction implies an increase in membrane area of 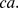 30% with respect its initial value (see Fig. 5b). Thus, this limiting case requires inexpensive membrane availability of the vesicle to increase its area [17]. This necessarily implies membrane uptake from either accumulated excess area or external lipid reservoirs [18], [19], [20], e.g. smaller vesicles or lipid aggregates in contact with the deformed vesicle. Otherwise, area expansion should occur at the expense of membrane stretching which is too expensive in terms of elastic energy [21]. Figure 6 shows the sequence of vesicle constriction occurred at constant volume. In this case, the vesicle with initial radius 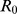 dilates its membrane area up to final fission in two identical spheres 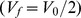 with smaller radius 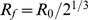. Constriction starts by breaking the spherical symmetry into an elongated shape (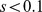). At 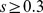, it evolves into a two lobed shape with a well defined spherocylindrical geometry characterised by an equatorial furrow with a saddle shape. Further constriction, (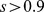), imposes a deeper furrow with a narrower neck between the two quasi-spherical lobes.

Constant area constriction is also a limiting case that deserves alternative discussion. A similar evolution of the vesicle profiles is observed in this case (data not shown). Such a constriction pathway requires the vesicle to decrease its initial volume forming two daughter specimens with a content smaller than the mother cell (see Fig. 5b), with 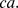 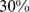 decrease of volume content. Indeed, the two final vesicles 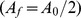 shrink to a volume smaller than the dimensions of the initial sphere, this is 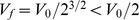. Lipid bilayers are indeed partially permeable to water [22], [23], thus, in the absence of active membrane pores, a constriction route at decreasing volume based in a partial loss of the water content may be easily envisaged.

### 2.2 Constriction force

**Figure 7:**
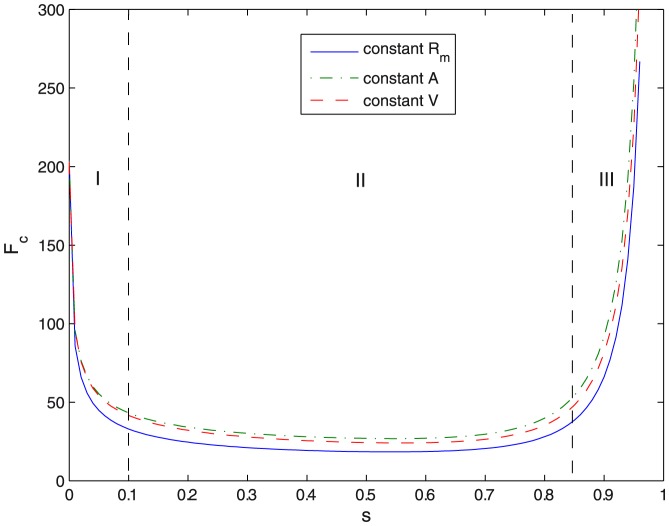
Constriction force. Constriction force 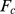 (in units of 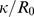) as a function of constriction parameter 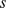. Due to its trend it is divided in three regimes (I, II and III) with different behaviour.

In terms of the change in bending energy, the constriction force is defined as
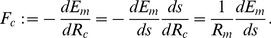
(16)This definition is valid for all cases (in particular for constant 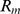, 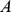 or 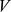), the difference arising from the functional dependence of 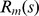 on the constriction ratio. Just because 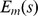 is scale invariant, we see from Eq. (16) that smaller vesicles are harder to constrict. Figure 7 shows the values of 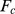 required for a given equatorial constriction to occur. This is calculated, following Eq. (16), as the numerical derivative of the minimal energy pathway in Fig. 4. No large differences are observed for constrained constriction along the three geometrical pathways considered, i.e. constant 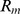, constant volume, and constant area. In all cases, three deformation regimes are clearly differentiated in Fig. 7. In the regime I at low constriction (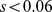) a kick-off force (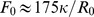) is required to initiate constriction from the initial spherical state. Thus, an initial force 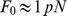 is required to initiate constriction deformations in cell sized vesicles 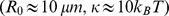. This is a value in the range of typical forces exerted by molecular motors [24], [25], [26], [27], [28]. Further elongation requires smaller forces. Once distorted, in the intermediate regime II (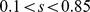), the vesicle becomes progressively elongated, plastic-like, under the action of a near constant force. Compared to the strong initial kick required for spherical distortion, a much weaker force is involved in such a plastic deformation (see regime II in Fig. 7). In the regime II, 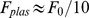, which causes a strong elongation followed by the formation of a constriction neck at the middle cell region (see profiles in Fig. 6). In the biologically relevant case, a constant force as small as 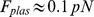 should be sufficient for making cell constriction to progress in this regime. From these results, we deduce that a strong force is required to break the initial spherical symmetry, however, once the symmetry is broken, the axially-elongated object is able to easily undergo the transitory shape transformations required to reach the pre-fissioned state. Finally, in the phase III, at high constriction (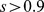), stronger forces are needed in order to overcome the curvature barrier involved in the pre-fissioned state (see Fig. 7). It is relevant to notice that the junction of the ansatzs for the poles and the constriction zone do not verify the matching conditions for higher derivatives [continuity of the second and third derivatives of 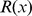], that the exact solution should verify. We expect these demanding conditions to be relevant to find a closer approximation to the exact shape at high constrictions, thus a better description of the curvature barrier preceding fission. However, preliminary numerical computations indicate that these improvements do not change the main quantitative conclusions of the present approach.

### 2.3 Stability of symmetrical constriction

The study of the energetics of symmetrical constriction is strongly motivated by its biological relevance. However, no less important is the question about its stability, a problem directly dealing with the propensity of liposomes to symmetrically divide in two daughter vesicles with a similar size. If symmetrical constriction was not stable, the question to know how large are the instabilities is a relevant problem with important implications in the chemical details of cell division. This is the question addressed in this section.

**Figure 8:**
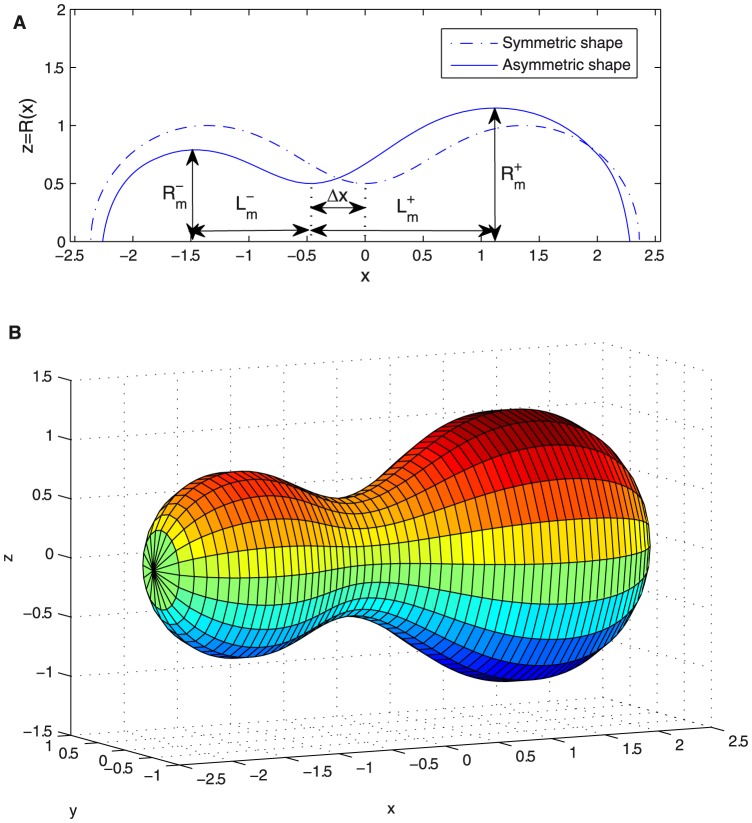
Asymmetrically constricted vesicle. A. Symmetric and asymmetric constriction optimal shapes [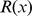 vs. 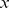] with 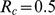 with the same volume plotted with the characteristic parameters for defining asymmetrical constrictions. B. Asymmetric surface resulting from the revolution along the 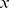 axis of the asymmetric 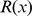 in Fig. 8A.

In previous sections, optimal membrane shapes have been considered for the case of symmetrical constriction. The variational approach has provided us with bending energies calculated in a broad range of constriction ratios defined under different geometrical restrictions. Here, we will consider the question of how stable the symmetrical constriction configuration is against longitudinal asymmetries. In order to perform this computation we compare the bending energy of a symmetrical shape with equal right and left lobes with an asymmetrical shape where one of the lobes is greater than the other (see Fig. 8). In the asymmetrical case, the constriction ratio 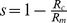 is different as seen from each one of the lobes, every one being characterized by a different 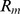 (see Fig. 8a). Consequently, for a given asymmetric configuration, both, optimal shape and bending energy are different for every lobe characterized by a different constriction ratio and size. In an asymmetric configuration, one of the lobes will have a greater maximum radius 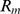 while the other will have an smaller one, with respect to the corresponding symmetric shape (see Fig. 8). We denote these changes as
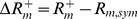
(17)for the righthand side, and
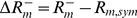
(18)for the lefthand side.

Analogous changes happen for the variable 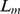 which now, in the asymmetric form, should be redefined as
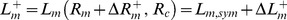
(19)for the righthand side, and
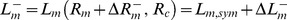
(20)for the lefthand side. These new parameters are clearly shown in Fig. 8a.

#### 2.3.1 Constant area

**Figure 9:**
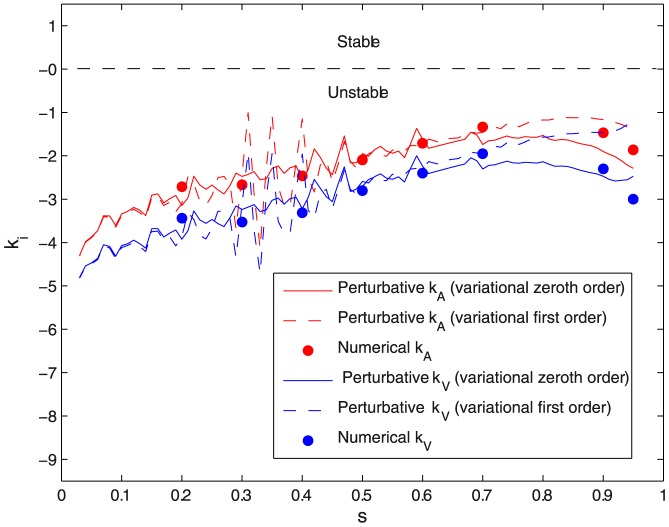
Instability coefficient of symmetrical constriction. Instability coefficient of symmetrical constriction for constant area 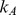 and for constant volume 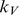 (in units of 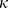) vs. constriction parameter 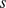 for different orders of approximation in the variational approach and calculated numerically.

If in the transition between the symmetric and the asymmetric shape the area is kept constant, the changes in 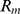 are related by
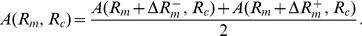
(21)which stands for the constant area of the asymmetrically deformed vesicle with the two lobes characterized by different 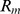 with respect to the undeformed case. This relation can be solved numerically and given 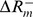 we can obtain the corresponding 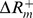, which maintains constant the area (see Fig. 9). Alternatively, for small departures from the symmetrical shape, an analytic perturbative computation is also possible. Expanding Eq. (21) up to second order, it is obtained:
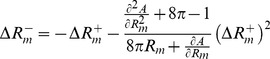
(22)With these values, one can already compute the bending energy 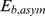 of the asymmetric shape, which is obtained as the sum of the bending energies of the two lobes considered individually:
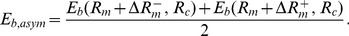
(23)We can also know how asymmetrical is the resulting shape, computing how much the constriction ring is displaced from the middle point between the poles (see Fig. 8):
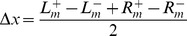
(24)Finally, for small 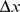, the difference of energy with respect to the symmetric configuration is given by a quadratic form as
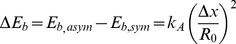
(25)with an effective harmonic constant 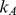


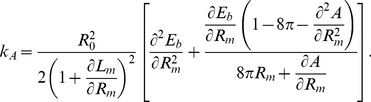
(26)


#### 2.3.2 Constant volume

If in the transition between the symmetric and the asymmetric shape it is the volume what is kept constant, the changes in 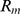 are related by
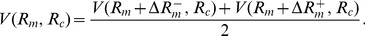
(27)As in the case of constant area we can solve this equation numerically and obtain 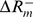 as a function of 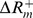 and compute 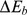 and 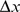 analogously (see Fig. 9). Alternatively, the perturbative computation is also possible. Assuming small asymmetries, one gets
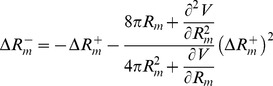
(28)implying an energy difference of
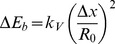
(29)with a constant volume effective harmonic constant
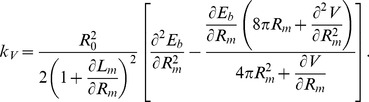
(30)


#### 2.3.3 Symmetric constriction is unstable

The harmonic constants in the quadratic forms in Eqs. (26) and (30) describe the effective change in elastic energy involved in an asymmetric constriction with respect to the symmetrical case. Figure 9 shows the values of these constants computed both, numerically and from the perturbative formulas in Eqs. (26) and (30). Similar results are found for the two cases: constant area and constant volume. Negative values of the harmonic constants are found in all cases, indicating lower bending energies in the asymmetric case than in the symmetric one, i.e. asymmetrical constriction is energetically more favourable than the symmetrical case of equatorial constriction. Within the considered harmonic approach, the larger the asymmetry the smaller is the penalty in elastic energy involved in constriction. In the two cases considered, symmetric constriction is systematically unstable with respect to the lateral displacement of the constriction neck, the case of constant volume being characterized by the highest decrease in elastic energy under asymmetric constriction 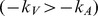. As expected, the highest instability is found for constriction at constant volume around the spherical geometry, indeed, the negative values of 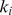 are expected to reach in this case a value 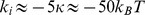 at 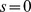. As contraction proceeds, the harmonic constants decrease in absolute value indicating a trend to a weaker destabilization with increasing constriction. A limiting value is reached at high constriction (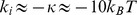 at 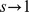), indicating the clear tendency of lipid vesicles to asymmetric budding instead of symmetrical constriction.

## 3 Conclusions

The mechanical problem of a spherical vesicle stressed under equatorial constriction was studied in the frame of the Helfrich-Canham Hamiltonian. The membrane shapes of minimal-energy were computed for vesicles deformed with a rotational symmetry using a variational approach. The bending energies were calculated as a function of symmetric constriction defining a continuous pathway between the undeformed sphere and the final prefission state. For negligible spontaneous curvature, membrane tension and osmotic stress, the bending energies show scale invariance. This is an important property which permits easy calculation of the constriction forces under different geometrical constraints, particularly constant radius, constant area, and constant volume. The constriction forces were computed, obtaining values in the range 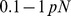 for cell-sized vesicles (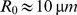) with a flexible membrane (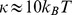). This defines cell constriction as a practicable deformation process under the action of cytokinetic engines based on sophisticated protein motors [24], [25], [26], [27] or simpler physical mechanisms taking advantage of phase segregation within the lipid component [29], [30]. Constriction at constant volume requires a nearly 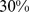 increase in area (see Fig. 5B), i.e., an intense membrane trafficking [31], [32], which is known to play an important role in cytokinesis [33], [34]. On the other hand if constriction takes place at constant area (i.e. without membrane trafficking) the volume must be reduced in 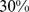 (see Fig. 5B). Thus, in constant area constriction a greater initial area is required to have the same final volume. Heat shock has been shown to increase the area before division [35], [36], and to affect membrane trafficking molecules genes expression, but also other genes as those of signaling molecules [37]. The other ideal case studied, constriction with constant maximum radius requires doubling the area and volume. Rod shaped cells present constrictions with constant maximum radius, but the rod shape reduces the required relative increase in area and volume with respect to spherical shaped cells [38], [39]. Another additional effect not included in our model is anisotropic contraction ring nucleation, which can lead to a non-concentric ring and break the axial symmetry [40].

The stability of the equatorial constriction was analyzed against lateral displacements of the deformation site. The energies of the asymmetric configurations were found smaller than the symmetric case corresponding to equatorial constriction. This indicates that symmetric division is unstable pointing out the functional requirement for a positioning mechanism that stabilizes the midcell emplacement of the constriction ring [41], [42], [43], [44], [45], [46]. We have only quantified here the instability arising from the bending energy minimization, cells also presents other instabilities, as for example those induced by the polar actomyosin contractility [28], [47]. These sources of instabilities are counterbalanced in cells by structures as the spindle apparatus and mechanism as bleb formation [39], [47], [48], [49], [50], [51].

The results constitute altogether a significant piece of knowledge on the physical mechanism of cell division through the mechanical pathways defined for optimal binary fission. The constriction pathway described here constitutes the simplest mechanism of symmetrical division of a spherical vesicle. Thus, it is expected to represent a minimal model for cell division by binary fission in primordial protocells [52], [53], [54]. In addition, as far as the essential mechanical features of such primordial division mechanism may be imprinted in more evolved cells, the results in this paper would serve to get insight on the more complex cytokinetic pathways involved in programmed division in modern cells [28], [55], [56], [57], [58].

The variational method used here can be used to obtain approximate analytical formulas to describe the general constitutive relations (work in progress).

## Supporting Information

Text S1 **Bending energy for surfaces of revolution.**
(PDF)
