# Supplementary material for: Mechanics of Constriction during Cell Division: A Variational Approach
Source: PLoS One. 2013 Aug 21;8(8):e69750. doi: 10.1371/journal.pone.0069750 (PMC3749217; doi:10.1371/journal.pone.0069750)
Supplement: Text S1 — Bending energy for surfaces of revolution. (PDF) [file pone.0069750.s001.pdf]

Supporting Information

Text S1: Bending energy for surfaces of revolution

The variations of energy due to the mean curvature in a general surface are given by

$$E_m = \frac{\kappa}{2}\int_{\Omega}[C_1(x,y)+C_2(x,y)]^2 dA. \tag{S1}$$

When the surface can be represented in Cartesian coordinates as

$$\vec{r} = (x, y, h(x,y))\,, \tag{S2}$$

the mean curvature is found to be given by

$$\frac{C_1+C_2}{2} = \frac{(1+h_x^2)h_{yy}+(1+h_y^2)h_{xx}-2h_xh_yh_{xy}}{2(1+h_x^2+h_y^2)^{3/2}} \tag{S3}$$

and the element of area by

$$dA = \sqrt{1+h_x^2+h_y^2}\; dxdy \tag{S4}$$

where the x and y subscripts represent partial derivatives with respect to this variable (see Ref. [3] for the derivation of the previous equations). As illustrative examples we can consider: a hemisphere of radius R_0 described by

$$h(x,y) = \sqrt{R_0^2-x^2-y^2}, \tag{S5}$$

which gives

$$\frac{C_1+C_2}{2} = -\frac{1}{R_0}, \qquad E_m^{(hemisph)} = 4\pi\kappa. \tag{S6}$$

and a hemicylinder of radius R_0 and length L described by

$$h(x,y) = \sqrt{R_0^2-y^2}, \tag{S7}$$

which gives

$$\frac{C_1+C_2}{2} = -\frac{1}{2R_0}, \qquad E_m^{(hemicyl)} = \pi\kappa\frac{L}{R_0} \tag{S8}$$

The scale invariance of these examples is in fact a general property of the bending Hamiltonian in Eq. (1). It can be shown that the bending energy E_m is invariant under the overall dilatation $x \to \lambda x$, $y \to \lambda y$, and $h \to \lambda h$. This implies that if we know the optimal shape for a given size, the optimal shape for other sizes can be obtained just by an overall rescaling.

For a surface of revolution with rotation symmetry axis along x,

$$h(x,y) = \sqrt{R^2(x)-y^2} \tag{S9}$$

parameterizes the upper half of the surface Ω_+ [the lower half of the surface Ω_- would be parameterized by $h(x,y) = -\sqrt{R^2(x)-y^2}$]. Using the parameterizations for $\Omega_{+/-}$ the mean curvature becomes

$$\frac{C_1+C_2}{2} = \frac{R_{xx}R-1-R_x^2}{2(1+R_x^2)^{3/2}R} \tag{S10}$$

Note that the results are independent on y, as corresponds to rotational symmetry around x. The element of area is

$$dA = R\sqrt{\frac{1+R_x^2}{R^2-y^2}}\; dxdy. \tag{S11}$$

If the surface is between x_i and x_f, its total area would be

$$A = 2\int_{\Omega_+} dA = 2\int_{x_i}^{x_f} R\sqrt{1+R_x^2}\int_{-R(x)}^{R(x)} \frac{1}{\sqrt{R^2-y^2}}\,dy\,dx$$
$$= 2\pi\int_{x_i}^{x_f} R\sqrt{1+R_x^2}dx, \tag{S12}$$

where the factor 2 outside the integral comes for the symmetry between Ω_+ and Ω_-, and we have integrated the y variable. For the total volume enclosed by the surface we have

$$V = 2\int_{\Omega_+} h(x,y)dydx = \pi\int_{x_i}^{x_f} R^2 dx. \tag{S13}$$

Analogously, once the membrane profile is known, the bending energy E_m [Eq. (S1)] for a surface of revolution is given by

$$E_m = \kappa\int_{x_i}^{x_f} \frac{(1+R_x^2-R_{xx}R)^2}{(1+R_x^2)^{5/2}R}\int_{-R(x)}^{R(x)} \frac{1}{\sqrt{R^2-y^2}}\,dy\,dx$$
$$= \pi\kappa\int_{x_i}^{x_f} \frac{(1+R_x^2-R_{xx}R)^2}{(1+R_x^2)^{5/2}R}dx. \tag{S14}$$

The scale invariance of the bending Hamiltonian in Eq. (S1) for surfaces of revolution implies no dependence of the bending energy on the system size. Thus, in Eq. (S14), for any shape the transformed under the overall dilatation $x \to \lambda x$ and $f \to \lambda R$ has the same bending energy. This overall dilatation transforms $R_x = \frac{dR}{dx}$ in $\frac{d(\lambda R)}{d(\lambda x)} = \frac{\lambda}{\lambda}\frac{dR}{dx}$, also $R_{xx}f$ in $\frac{d}{d(\lambda x)}\left[\frac{d(\lambda R)}{d(\lambda x)}\right]\lambda R = \frac{\lambda^2}{\lambda^2}\frac{d^2R}{dx^2}R = R_{xx}f$, and $\frac{dx}{R}$ in $\frac{d(\lambda x)}{\lambda R} = \frac{\lambda}{\lambda}\frac{dx}{R} = \frac{dx}{R}$. Therefore, the whole expression for the bending energy is scale invariant. Thus, once we have determined the shape that minimizes the energy, its transformed under an overall dilatation has the same energy and also minimizes the energy. Analogously it can be shown that under an overall dilatation the area is transformed as $A \to \lambda^2 A$ and the volume as $V \to \lambda^3 V$.
